# Association of Gender with Survival in Melanoma In Situ of the Head and Neck: A National Database Study

**DOI:** 10.7759/cureus.6924

**Published:** 2020-02-09

**Authors:** Vignesh Ramachandran, Asad Loya, Kevin Phan

**Affiliations:** 1 Dermatology, Baylor College of Medicine, Houston, USA; 2 Medicine, Baylor College of Medicine, Houston, USA; 3 Dermatology, Liverpool Hospital, Sydney, AUS

**Keywords:** melanoma in situ, head and neck, survival, gender, association, database, seer, otolaryngology, mis, dermatology

## Abstract

Introduction

While prior studies have addressed the gender-specific survival of malignant melanoma, such investigation is lacking for melanoma in situ (MIS) and for the sun-exposed head and neck areas. Understanding the role of patient characteristics on disease prognosis is essential in determining optimal patient treatment and follow-up. We conducted a retrospective cohort study of patients diagnosed with MIS of the head and neck to assess the association of gender with long-term survival.

Methods

First primary cases of MIS diagnosed between 1998 and 2015 were extracted from the National Cancer Institute’s Surveillance, Epidemiology, and End Results (SEER) database. Cox regression analysis adjusting for demographic, tumor, and treatment characteristics was used to evaluate all-cause and cancer-specific mortality risks.

Results

After adjusting for demographic, tumor, and treatment data, males demonstrated significantly poorer overall survival (hazard ratio [HR] 1.484; 95% confidence interval [CI] 1.332, 1.653; P<0.001) and cancer-specific survival (HR 1.571; 95% CI 1.056, 2.338; P=0.026) compared to their female counterparts.

Conclusion

Proposed reasons for these findings include gender-based hormonal influence on cancer growth and development, gender-specific health utilization behaviors, and gender-based cosmetic impact of cutaneous malignancies. These findings do have limitations, including its retrospective nature, possible upgrading of MIS diagnoses during the study period, miscoding, and inability to account of lifestyle/modifiable/environmental risk factors. Nevertheless, it suggests a gender-specific survival difference, which may be further investigated and considered as part of clinician awareness, influence patient counseling, and screening for such patients.

## Introduction

Melanoma is a potentially fatal form of skin cancer and its prognosis sharply declines from in situ (stage 0) to stage 4 [1]. Current research focuses on prognostic factors and associations related to survival in melanoma, but such literature is often lacking for melanoma in situ (MIS) [2]. Such studies have documented the gender-specific survival advantage of females in early stage (stage 1) melanoma [3,4]. However, such investigation is lacking for MIS and for melanoma of the head and neck, the most common presenting location of melanomas [5]. Herein, we utilize the National Cancer Institute’s Surveillance, Epidemiology, and End Results (SEER) database to investigate the association of gender with survival in patients with MIS of the head and neck.

## Materials and methods

We conducted a retrospective analysis using the SEER database, which provides data on approximately 34.6% of the United States population [6]. Primary cases of MIS (1998-2015) were identified using the International Classification of Diseases for Oncology Third Edition (ICD-O-3) histological codes for MIS (8,720/2), MIS regressing (8,723/2), nodular MIS (8,721/2), balloon cell MIS (8,722/2), amelanotic MIS (8,730/2), MIS in junctional nevus (8,740/2), superficial spreading MIS (8,743/2), acral lentiginous MIS (8,744/2), desmoplastic MIS (8,745/2), mucosal lentiginous MIS (8,746/2), MIS in compound nevus (8,760/2), MIS in giant pigmented cell (8,761/2), epithelioid and spindle cell MIS (8,770/2), epithelioid cell MIS (8,771/2), and spindle cell MIS (8,772/2). Cases were extracted without "malignant" field selected in the SEER database with the designation "/2," which reflects the following characteristics as per the SEER morphology classification: carcinoma in situ, intraepithelial, noninfiltrating, or noninvasive. ICD-O-3 topographical codes were further used to limit cases to the head and neck region, specifically as face and scalp or neck [7]. Demographic, tumor, treatment, and survival characteristics were extracted. Univariate and multivariate Cox regression analyses were conducted to determine all-cause and cancer-specific mortality risks. SPSS software (version 25, IBM Corp., Armonk, NY) was used to conduct all statistical analyses. A P-value of <0.05 was used to determine statistical significance.

## Results

A total of 9,881 cases of MIS of the head and neck were identified over a mean follow-up period of 76.6 (±57.0) months. Additionally, 68.7% (6,787/9,881) of the overall cohort were male. Demographic, tumor, and treatment characteristics are displayed in Table 1. The 10-year overall survival (OS) was 79% for females, compared to 74% for males. After adjusting for demographic, tumor, and treatment data (found in Table 1), male subjects demonstrated significantly poorer OS (hazard ratio [HR] 1.484; 95% confidence interval [CI] 1.332, 1.653; P<0.001) and cancer-specific survival (HR 1.571; 95% CI 1.056, 2.338; P=0.026) compared to their female counterparts (Table 2). The female subgroup is the reference group given its higher 10-year survival; thus, it is used as the reference for the Cox regression analysis, which necessitates comparison to a reference group to compute HRs for unadjusted and adjusted survival in males.

**Table 1:** Demographic, tumor, and treatment characteristics of patients with melanoma in situ of the head and neck regions. *p < 0.05. MMS, Mohs micrographic surgery; WLE, wide local excision, LN, lymph node.

		Sex						
		Female		Male		Total		Chi-square
		N	%	N	%	N	%	P-value
Age group	0-64 years	1429	46.2%	3066	45.2%	4495	45.5%	
	65-74 years	731	23.6%	1845	27.2%	2576	26.1%	
	75+ years	934	30.2%	1876	27.6%	2810	28.4%	
Race	White	2803	90.6%	6206	91.4%	9009	91.2%	0.010*
	Black	7	0.2%	11	0.2%	18	0.2%	
	Other	25	0.8%	22	0.3%	47	0.5%	
	Unknown	259	8.4%	548	8.1%	807	8.2%	
Hispanic ethnicity	Non-Hispanic	2981	96.3%	6647	97.9%	9628	97.4%	
	Hispanic	113	3.7%	140	2.1%	253	2.6%	
Marital status at diagnosis	Married or domestic partner	1268	41.0%	3659	53.9%	4927	49.9%	
	Single (never married)	270	8.7%	476	7.0%	746	7.5%	
	Divorced‎/separated	199	6.4%	201	3.0%	400	4.0%	
	Widowed	390	12.6%	203	3.0%	593	6.0%	
	Unknown	967	31.3%	2248	33.1%	3215	32.5%	
Reporting source	Hospital inpatient‎/outpatient‎/clinic	1685	54.5%	3634	53.6%	5319	53.9%	0.691
	Physicians office‎/private medical practitioner	989	32.0%	2177	32.1%	3166	32.1%	
	Laboratory only	384	12.4%	889	13.1%	1273	12.9%	
	Other	34	1.1%	84	1.2%	118	1.2%	
Primary site	Face	2552	82.5%	4928	72.6%	7480	75.7%	
	Scalp‎/neck	542	17.5%	1859	27.4%	2401	24.3%	
Laterality	Unilateral	2450	79.2%	5049	74.4%	7499	75.9%	
	Bilateral	5	0.2%	14	0.2%	19	0.2%	
	Not a paired site	447	14.4%	1380	20.3%	1827	18.5%	
	Midline	40	1.3%	79	1.2%	119	1.2%	
	Unknown	152	4.9%	265	3.9%	417	4.2%	
Diagnostic confirmation	Positive histology	3075	99.4%	6744	99.4%	9819	99.4%	0.909
	Other	19	0.6%	43	0.6%	62	0.6%	
Surgery type	WLE	1657	53.6%	3696	54.5%	5353	54.2%	0.680
	MMS	823	26.6%	1757	25.9%	2580	26.1%	
	No surgery	614	19.8%	1334	19.7%	1948	19.7%	
LN biopsy	LN removed	3	0.1%	16	0.2%	19	0.2%	0.179
	Sentinel LN biopsy	8	0.3%	17	0.3%	25	0.3%	
	Both	1	0.0%	3	0.0%	4	0.0%	
	None	2396	77.4%	5369	79.1%	7765	78.6%	
	Unknown‎/NA	686	22.2%	1382	20.4%	2068	20.9%	
Tumor size	<1 cm	627	20.3%	1385	20.4%	2012	20.4%	0.985
	>1 cm	8	0.3%	17	0.3%	25	0.3%	
	Unknown	2459	79.5%	5385	79.3%	7844	79.4%	
Tumor ulceration	Not present	1962	63.4%	4352	64.1%	6314	63.9%	0.326
	Present	8	0.3%	29	0.4%	37	0.4%	
	Unknown‎	1124	36.3%	2406	35.5%	3530	35.7%	

**Table 2:** Unadjusted and adjusted Cox regression analysis.

		Overall survival		Cause-specific survival	
		HR (95% CI)	P-value	HR (95% CI)	P-value
Unadjusted analysis	Female	Reference	Reference	Reference	Reference
	Male	1.215 (1.098, 1.344)	<0.001	1.401 (0.964, 2.037)	0.077
Adjusted analysis	Female	Reference	Reference	Reference	Reference
	Male	1.484 (1.332, 1.653)	<0.001	1.571 (1.056, 2.338)	0.026

## Discussion

Improved melanoma survival for females has been reported in a large clinical trial and in European population-based studies [8,9]. However, these findings, to our knowledge, have yet to be investigated for MIS. In our study, we focus on the most common location of MIS (head and neck) in order to report clinically relevant findings. In doing so, we demonstrate that male patients have worsened melanoma-specific survival for MIS of the head and neck. Several mechanisms may account for this finding.

In their population-based study of 11,734 melanoma patients, Joosse et al. revealed a significant female advantage for melanoma-specific survival, lower risk of cancer progression (including reduced risk of lymph node and visceral metastases), and survival advantage even after first progression, although another study found no difference in survival between males and females with regards to metastatic disease [4,8]. To this end, they discussed the possibility of female gender-related factors that may portend a survival advantage or males may demonstrate a melanoma-stimulating factor [8]. Similarly, Molife et al. suggested that gender may influence distinct factors implicated in phases of disease in melanoma, primarily local primary tumor invasion [10]. Female steroids decrease invasion of melanoma cell lines through fibronectin, whereas male and adrenal steroids do not [11,12]. This may influence progression from MIS to higher stage melanoma which would result in worsened prognosis and survival.

Additionally, males, compared to females, are less likely to self-detect melanomas, have lower perceptions of skin cancer risk, and are less often utilize healthcare providers [1]. MIS represents an early detectable disease state that is readily-treated if care is sought early enough through self-detection or surveillance by a healthcare provider [13,14]. As such, intrinsic healthcare behaviors factors may portend worsened survival in males with MIS serving as a surrogate for possibly other cancers at large [14]. This may explain the worse male survival in seven different common malignancies presented by Molife et al. [10]. Furthermore, our study assessed a cosmetically sensitive region-the head and neck. Al-Dujaili et al. demonstrated that women pay close attention to skin health, especially of the face, which is where skin cancers often manifest [15]. This may lead to earlier detection and translate to cancer care-related behaviors portending improved outcomes

## Conclusions

The limitations of our study include its retrospective nature, possible upgrading of MIS diagnoses during the study period, miscoding, and inability to account of lifestyle, modifiable, and environmental risk factors. While cases were extracted strictly by SEER morphology codes, upgrading of malignancies could be possible and not accounted for during the time period of the survival analysis. Additionally, clinical practices vary by hospitals and facilities reporting to SEER. For instance, sentinal lymph node biopsy was performed for a small subset of our overall cohort. This practice is not commonplace for MIS disease. Nevertheless, this variable was included as part of our multivariate analysis along with a plethora of other variables in order to account for as many factors as possible.

Overall, MIS represents the earliest form of melanoma (stage 0), so identifying prognostic markers such as gender-specific differences in survival may heighten clinician awareness, influence patient counseling, and screening. Future studies would be well intended in identifying ways to mitigate gender-based disparities in melanoma survival.
